# Vaccines: a promising therapy for myelodysplastic syndrome

**DOI:** 10.1186/s13045-023-01523-4

**Published:** 2024-01-08

**Authors:** Kriti Gera, Anjali Chauhan, Paul Castillo, Maryam Rahman, Akash Mathavan, Akshay Mathavan, Elizabeth Oganda-Rivas, Leighton Elliott, John R. Wingard, Elias J. Sayour

**Affiliations:** 1 Department of Medicine, University of Florida, Gainesville, FL USA; 2 Department of Neurosurgery, Preston A. Wells, Jr. Center for Brain Tumor Immunotherapy, University of Florida, Gainesville, FL USA; 3 Division of Hematology and Oncology, Department of Pediatrics, University of Florida, Gainesville, FL USA; 4 Division of Hematology and Oncology, Department of Medicine, University of Florida, Gainesville, FL USA

**Keywords:** Myelodysplastic neoplasms, Immunotherapy, Vaccines, Dendritic cells

## Abstract

Myelodysplastic neoplasms (MDS) define clonal hematopoietic malignancies characterized by heterogeneous mutational and clinical spectra typically seen in the elderly. Curative treatment entails allogeneic hematopoietic stem cell transplant, which is often not a feasible option due to older age and significant comorbidities. Immunotherapy has the cytotoxic capacity to elicit tumor-specific killing with long-term immunological memory. While a number of platforms have emerged, therapeutic vaccination presents as an appealing strategy for MDS given its promising safety profile and amenability for commercialization. Several preclinical and clinical trials have investigated the efficacy of vaccines in MDS; these include peptide vaccines targeting tumor antigens, whole cell-based vaccines and dendritic cell-based vaccines. These therapeutic vaccines have shown acceptable safety profiles, but consistent clinical responses remain elusive despite robust immunological reactions. Combining vaccines with immunotherapeutic agents holds promise and requires further investigation. Herein, we highlight therapeutic vaccine trials while reviewing challenges and future directions of successful vaccination strategies in MDS.

## Introduction

Myelodysplastic neoplasms (MDS) are a diverse collection of hematopoietic neoplasms characterized by inadequate hematopoiesis, dysplasia and pancytopenia. It is a disease seen in  elderly patients at median diagnostic age of  77 years [1]. The management is complex due to a wide range of etiologies, presentations and clinical courses. MDS is divided into low, intermediate-1, intermediate-2 and high risk (HR-MDS) based on risk stratification according to the Revised International Prognostic Scoring System (IPSS) [2]. Low-risk MDS (LR-MDS) is characterized by an indolent course with a median survival of 3–10 years. In contrast, HR-MDS is associated with profound cytopenias and rapid evolution to acute myeloid leukemia (AML) and has a median survival of only 5–14 months [3].

Over 100 genes are implicated in MDS encoding for the spliceosome, and chromatin, epigenetic and transcriptional modulators. The most common high-risk mutations are ASXL1, RUNX1, TP53, EZH2, ETV6 and SF3B1K666N [4]. The mutational landscape is dynamic over the course of disease which further complicates management and clinical decision-making. Significant immune abnormalities are observed in MDS patients [5]. In low-risk MDS (LR-MDS), there is an activated immune state, characterized by increased cytotoxic T lymphocytes (CTL) and helper T cell 17 (Th17), while regulatory T cells (Treg) decrease [5]. On the other hand, high-risk MDS (HR-MDS) is associated with immune inhibition, leading to the proliferation of abnormal clones in the bone marrow microenvironment. Immunotherapy may overcome these complex immune abnormalities in MDS and has emerged as a treatment option [6].

Current drug treatments include hypomethylating agents (HMAs) such as azacitidine (AZA) and decitabine, low-dose cytarabine and lenalidomide (5q deletion). Recently, improvement in the understanding of MDS biology has led to the approval of luspatercept for SF3B1 mutated MDS [7]. Allogeneic hematopoietic stem cell transplant (alloHCT) is the only curative option, but is often limited by old age and fitness criteria. There are currently no established treatment options available for elderly patients who do not respond to HMA therapies. Consequently, there is a dire need for new investigational agents. Several randomized phase III trials of novel therapies targeting immunological and epigenetic processes are currently underway, but none has been approved to date [6]. While immune checkpoint inhibitors are approved for solid tumors and lymphoma, they have shown limited efficacy in myeloid malignancies [8, 9].

Over the last decade, cancer vaccines have emerged as systemic treatment option particularly for patients who cannot tolerate toxic chemotherapy [10]. To date, the US FDA has granted approval to just one therapeutic cancer vaccine, for prostate cancer, sipuleucel-T, which increases patient survival by only four months [11]. Recently, two separate initiatives using personalized mRNA vaccines have shown promising results. In February 2023, the FDA granted breakthrough designation for a combination of a personalized mRNA vaccine (mRNA-4157/V940) and pembrolizumab for high-risk resected melanoma, showing a 44% higher recurrence-free survival compared to pembrolizumab alone in phase 2b KEYNOTE-942 trial [12]. In May 2023, a phase 1 trial reported that patients with resected pancreatic cancer treated with chemotherapy, atezolizumab and a personalized mRNA vaccine had a 50% cancer-free rate [13]. Cancer vaccines are designed to generate anti-tumor immunity by stimulating cancer-specific T-cells, but often remain limited by weak immunogenicity, antigen presentation and/or ability to escape immunosurveillance by tumor cells, which are significant hurdles to immunotherapeutic efficacy in MDS [14]. Among the hematological malignancies, there is a notable paucity of vaccine development studies for MDS. A challenge faced in preclinical studies is to replicate the complex molecular and clinical behavior of human MDS in animal models [15]. The clonal heterogeneity, molecular evolution coupled with emergence of resistant clones further complicates selection of therapeutic targets [16], [17]. Tables 1 and 2 provide an overview of completed and ongoing cancer vaccine trials in MDS respectively. In this review, we will discuss contemporary vaccine therapies for MDS including challenges and future directions.

## AML and MDS: shared origin, divergent genetics and implications

AML and MDS represent a continuous spectrum of myeloid malignancies, arising from a common biological origin with overlapping clinical features but differing in genetic composition [18]. Approximately 20–30% of MDS cases progress to AML, while 30% of AML cases (secondary AML) emerge from prior hematological conditions such as MDS [19]. In the past, distinction between these two entities was primarily based on blast percentage and clinical presentation. However, our deepening knowledge of the genetic features of these diseases has become an important factor in distinguishing and managing them. For instance, mutations in genes such as TP53, EZH2, RUX1, U2AF1 and ASXL1 in MDS signify a higher risk of transformation to AML [20]. A study revealed a positive correlation between WT-mRNA expression in MDS and the likelihood of progression to AML [21]. This growing understanding has prompted several clinical trials investigating drugs targeting common driver mutations to include patients with both AML and MDS to ensure a comprehensive study cohort. However, it is also crucial to analyze outcomes separately to ensure the unique characteristics and needs of each group are adequately addressed.

## Peptide vaccines

Expressed tumor/leukemia-associated antigens (TAAs or LAAs) can be synthesized as peptide vaccines to generate immunological response *in vivo*. An ideal LAA immunotherapeutic target would be immunogenic, specific, nontoxic and pivotal in tumor biology [1, 8].

The major challenge in identifying suitable antigen targets for MDS lies in their low immunogenicity and specificity coupled with their genetic instability. In MDS, an initial dominant clone with specific mutations drives disease manifestation, but genetic instability leads to emerging subclones carrying new mutations; these expressed antigens can outcompete dominant clones, posing a challenge to identification and specification of targetable antigens. Since malignant evolution in MDS is heterogenous with lower subclonal fractions relative to the initial dominant clone, there remains limited success in many vaccination strategies and immunotherapies [22]. Although these challenges exist, there is growing evidence indicating clinical effectiveness of peptide vaccines in MDS, especially in those with low disease burden [14].

The efficacy of peptide vaccines depends on several factors, including activation of antigen-presenting cells (APCs), peptide affinity, peptide length, systemic spread, antigenicity of adjuvants and mode of administration. In general, longer antigen epitope structure provides coverage for high HLA polymorphisms in the general population, stability from enzymatic degradation and multiple sites to elicit immunogenic response; however, cross-reactivity might be limiting [23] and repeated doses of peptide vaccines might elicit immune tolerance due to T cell anergy and over stimulated regulatory T-cells [24]. For instance, repeated injections of vaccines resulted in loss of immune response [24]. To avoid this, some studies have suggested using peptides derived from two different leukemia-associated antigens.

**Fig. 1:**
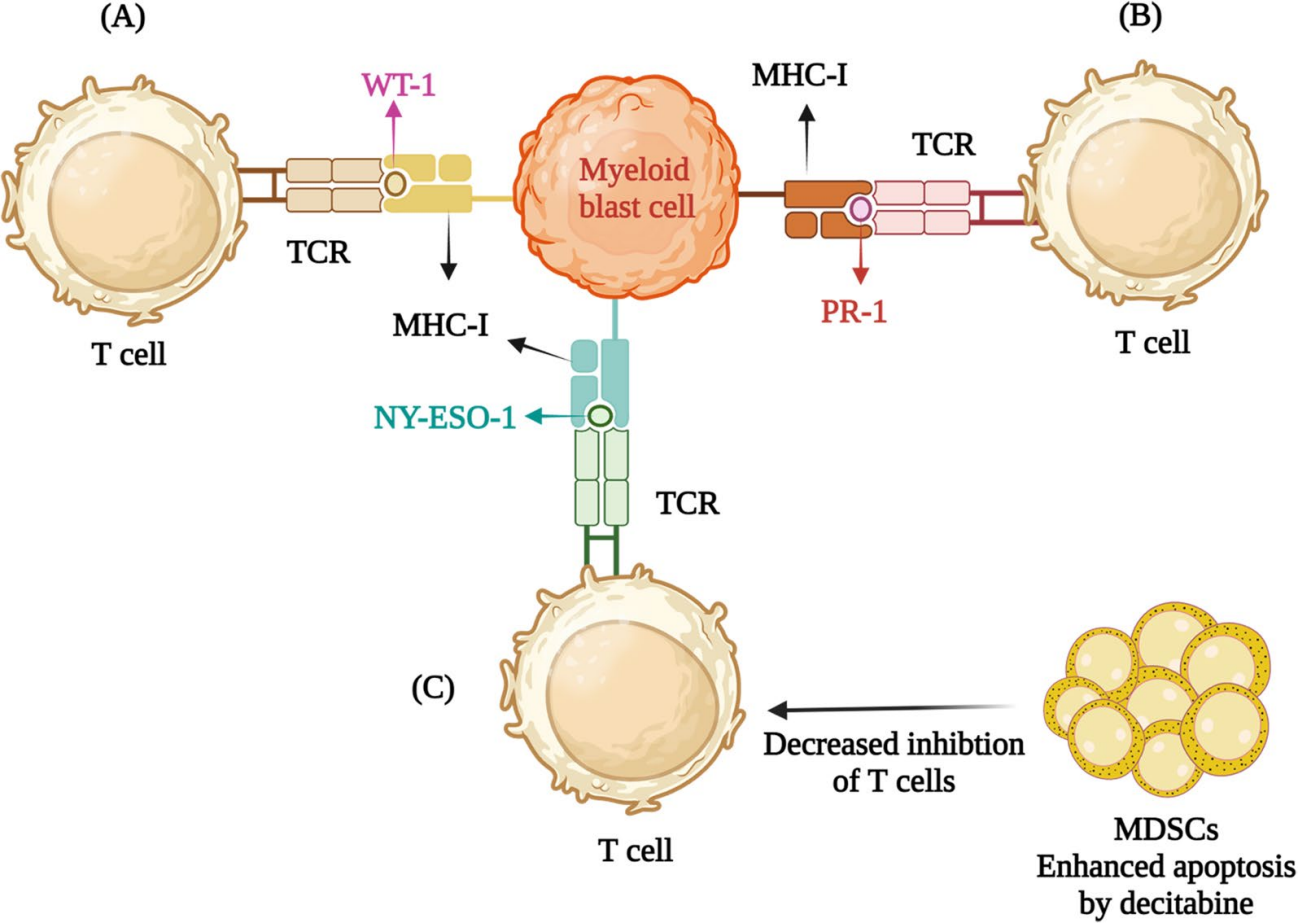
Peptide vaccine targets for MDS. **A** WT-1 (Wilms tumor 1) peptide is a zinc finger transcription factor overexpressed in leukemic blasts in MDS and is associated with poor prognosis. **B** PR-1 is a human leukocyte HLA-A2 restricted peptide derived from the azurophil granule proteases neutrophil elastase (NE) and proteinase-3 (P3) and is highly expressed on myeloid blasts cells. **C** NY-ESO-1 is a cancer testis antigen (CTA) and its expression is upregulated on myeloid cells after treatment with hypomethylating agents (HMAs) such as decitabine

Several TAAs or LAAs have been explored in MDS. The majority of them are shared antigens with other hematological malignancies, especially AML. Among various peptide vaccines, the notable targets are Wilms’ tumor 1 (WT-1) antigen, proteinase-3+ neutrophil elastase (PR-1), NY-ESO-1 peptide, preferentially expressed antigen of melanoma (PRAME) and receptor for hyaluronic acid-mediated motility (RHAMM) (Fig. 1).

### Wilms tumor 1 (WT-1)

The WT-1 gene is located on chromosome 11q13 and encodes factors that play key roles in cell growth and differentiation [25]. WT-1 can mediate oncogenesis, is highly expressed on blasts and confers a poor prognosis when present in MDS [26]. One study investigating extent of WT-1 expression in various IPSS groups of MDS identified a significantly greater expression of WT-1 with a median of 2,262 (range 227–11,006) copies in RAEB (refractory anemia with excess blasts) compared to a median of 4 (range 1–22) in healthy controls [27]. WT-1 expression was significantly greater in RAEB when compared with refractory anemia (RA) [27]. Compared with historical controls, clinical trials targeting WT-1 have improved relapse-free survival and transfusion independence in AML and high-risk MDS, respectively [28]. Pilot trials using WT-1 peptide vaccines have shown the ability to stimulate a clonal proliferation of WT-1 reactive T cells, which are primarily CD8+T cells [29].

Expanding WT-1 reactive T cells from major histocompatibility complex (MHC)-I or II bound peptides did not elicit adverse effects or autoreactivity in reported studies to date. WT4869 is a peptide vaccine consisting of an epitope derived from WT-1 protein product restricted to HLA-A*24:02 (common in the Japanese population), and in a phase 1 clinical trial, WT-1-specific T cell responses were observed in 11 of the 25 patients with MDS evaluated in the study [30]. Adverse reactions were seen in 22 patients (85%), with 6 patients (23.1%) discontinuing therapy due to intolerance, but most adverse events were manageable [30]. Another WT-1 vaccine, DSP-7888, consisting of two different WT-1-derived epitopes, for intradermal administration was tested by administration every 2 weeks (for 6 months) in 12 patients with HR-MDS who failed AZA therapy; no dose limiting toxicities were observed and only 2.1% patients (1 event) had ≥ grade 4 toxicity [31]. Subsequently, a phase 2 trial was conducted in 35 additional patients where the median OS was 8.6 months greater that the historically reported median OS of 5.6 months; approximately 80% of the patients showed WT-1-specific immunological responses [31]. Paradoxically, the median OS was unexpectedly lower compared to WT4869, which may be attributed to sampling as the survival outcomes for WT4869 were drawn from a subgroup analysis with a small sample size of only 11 patients. In a review of 9 WT-1 vaccine trials in AML and MDS, there were no reported grade 3 or 4 toxicities in 8 of those trials [32]. These 9 clinical trials showed some clinical responses including four MDS patients maintaining stable disease and two MDS patients displaying significant neutrophilic responses [24, 29, 33]. These findings support the promise of WT-1 as a safe and effective immunotherapeutic target in MDS that bears promise for further development.

### Proteinase-3+ neutrophil elastase (PR-1)

PR-1 is an HLA-A2-restricted peptide from protein proteinase-3 (P3) and neutrophil elastase 3 (NE) and it is found in elevated concentrations within the primary granules of myeloid blasts in MDS [34]. PR-3-specific immune responses have been observed in HLA-A2 positive MDS patients; however, persistent overexpression may result in immunological tolerance and T cell anergy [34], [35]. A study involving 66 patients with AML, MDS and CML who received a PR-1 vaccine demonstrated PR-1-specific T-cell response in 53% of patients [36]. The clinical response correlated with the level of disease burden. Moreover, there were clinical responses in 4 of 11 patients with MDS, one patient with partial remission and 3 with HI (hematological improvement) [36]. There were no reported grade 3 and higher toxicities. The clinical responses were largely seen in patients with lower disease burden. Similar to the WT-1 vaccine, the PR-1 vaccine has been found to be safe, effective and displays a potential therapeutic role in settings of low tumor burden including low-risk MDS, or as consolidation therapy in higher-risk MDS [36].

### Combined WT-1 and PR-1 vaccine

The combination of PR-1 and WT-1 antigen-specific vaccine, designed to enhance the antigenic targeting was investigated in a clinical trial for AML/MDS patients [24, 29]. A total of 8 patients received the vaccine of whom 2 patients had MDS (refractory anemia, refractory anemia with ringed sideroblasts). Both MDS patients had a PR-1- or WT-1-specific CD8+ T-cell response and experienced only grade 1 toxicities [24, 29] While repeated vaccination led to preferential proliferation of low-avidity CD8+ T-cell and loss of vaccine immunogenicity, anti-leukemic activity detected by reduction in WT-1 transcripts correlated positively with the presence of high-avidity CD8+ T cells in two patients and both patients had stable disease for > 2 years [24, 29].

### NY-ESO-1

Cancer testis antigens (CTAs) are a highly immunogenic family of antigens expressed in solid cancers [37], but tend to have silent expression in MDS as a byproduct of promotor hypermethylation [38]. Although their mechanism of action remains unclear, hypomethylating agents are often used as front-line therapies with a propensity to elicit CTA expression that can be immunotherapeutically exploited [39]. While CTAs like NY-ESO-1 can be expressed in germline tissue, these tissues tend to be devoid of MHC-I expression allowing CD8+ T-cells to be uniquely tumor-specific [40].

A vaccine that targets NY-ESO-1 has a strong safety profile and capacity to generate robust T-lymphocyte mediated cytotoxic responses in preclinical studies [41]. A phase 1 human clinical trial included 7 patients with intermediate- or high-risk MDS who received the NY-ESO-1 peptide vaccine in combination with decitabine therapy for 4 cycles [42]. All patients displayed NY-ESO-1 expression on myeloid cells; CD4+ and CD8+ T-cell responses were observed in 6 and 4 patients, respectively, and the vaccine response was associated with an increased frequency of activated dendritic cells detected by flow cytometry [42]. While the trial established safety and efficacy in this group, the immunological response was weaker than seen in trials targeting NY-ESO-1 in solid tumors [43, 44]. To increase efficacy, a phase 1 clinical trial was conducted with NY-ESO-1 fusion protein in combination with decitabine and nivolumab (DEC-205/NY-ESO-1) [45]. The results of this trial have yet to be reported.

Another study evaluated a novel multiantigen vaccine targeting NY-ESO-1, MAGE-A3 (Melanoma antigen family A), PRAME and WT-1 in combination with azacitidine therapy in a study for HR-MDS [46]. Unfortunately, all patients (*n* = 5) progressed to AML and the study was terminated [46]. Immune responses were not identified by intracellular cytokine staining or ELISpot assays, but changes in the expression of immune-specific stimulatory and inhibitory markers were observed. Only 1 patient demonstrated grade 4 toxicity (neutropenia requiring antibiotic prophylaxis) [46]. To date, no trials have tested this strategy in lower-risk MDS or earlier in the course of MDS in which there would be greater time to test for more robust responses.

### Receptor for hyaluronic acid–mediated motility (RHAMM)

RHAMM is a cell surface receptor expressed in tumor cells in AML, MDS, CML, CLL (chronic lymphocytic leukemia) and MM (multiple myeloma) patients [47]. The biological role of this receptor is to facilitate generation of a cell cycle protein involved in microtubular stability and cell migration [47]. Upregulation in cancer cells can lead to metastasis and rapid proliferation of cancer cells. Schmitt et al. developed a RHAMM-derived CD8+ T cell epitope which was able to elicit cytotoxic T cell responses against myeloid blasts [48]. In a phase 1 trial, 3 MDS patients (RA, RAEB) received 4 vaccination doses in a biweekly schedule [48]. One patient attained significant reduction of blasts and one patient became transfusion-independent after 4 doses. Unfortunately, subsequent trials did not show improvement in immunological responses with higher dosages [49]. Shortly after, Snauwaert et al. showed that the expression of RHAMM on leukemia stem cells was similar to that of hematopoietic cells in healthy controls [50].

### Diphtheria toxin fusion protein

A novel SL-401 (DLT388IL-3) prepared by integrating catalytic and translocation domains of diphtheria toxin (DT388) with interleukin 3 (IL-3) elicited immunological responses against myeloid stem cells in in vitro and in vivo studies [51]. In a phase I/II clinical trial in 31 patients with AML (median age 62 years), including four patients with antecedent MDS, patients were treated with escalating doses of DT388IL-3. Complete response (CR) of 8-month duration was observed in one patient [52]. Two patients demonstrated partial responses (PR) and three patients had minimal responses with clearance of peripheral blasts along with a decrease in marrow blasts. Responses were limited to patients without prolonged myelosuppression [52]. A phase 1 clinical trial evaluating SL-401 in combination with azacitidine or azacitidine/venetoclax in AML, HR-MDS and BPDCN is currently ongoing [53].

In conclusion, peptide vaccination has been found to be a safe treatment strategy for MDS patients with few dose limiting toxicities. However, substantial clinical benefit has yet to be established. Likely major limitations are the immunosuppressive mechanisms, including regulatory cells in the tumor microenvironment. Improving peptide vaccination outcomes relies on addressing these immune regulatory mechanisms. One such approach, as described above, is combining HMA with CTA-targeted vaccines, which not only enhances CTA expression on tumor cells but also reduces myeloid-derived suppressor cells (MDSCs) proliferation in the bone marrow [54]. Furthermore, the variation in responses to peptide vaccines likely arises from multiple factors, including the patient's immune status before immunization and whether tested early or late in the course of the disease progression. It is crucial to prioritize the development and validation of assays for assessing and predicting a patient's responsiveness to a tumor vaccine.

Peptide vaccines, particularly the ones using HLA class I restricted peptides have encountered challenges in generating strong CD8+ T cell responses due to HLA diversity and suboptimal peptide binding. Consequently, long-term anti-MDS and anti-leukemic effects have been limited following repeated vaccination [29] and newer studies are using peptide antigens that are recognized by both MHC-I and MHC-II [55]. Additional research is required to determine if such modifications will result in more robust and long-lasting immunity.

## Whole-cell vaccines

**Fig. 2:**
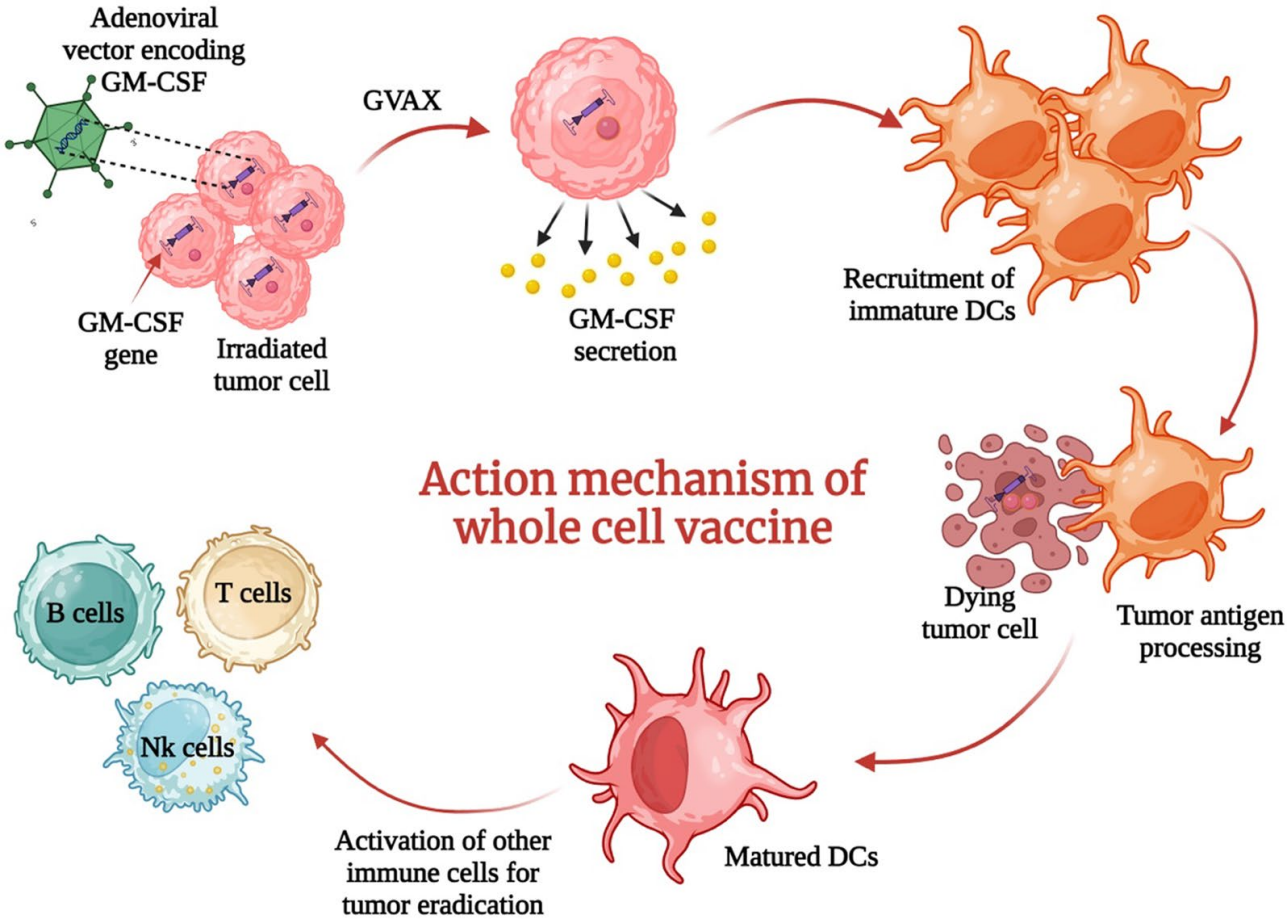
Schematic figure showing preparation and mechanism of action of GVAX whole-cell vaccine

Preclinical studies in the 1990s demonstrated that irradiated tumor cells were not effective in generating anti-tumor immunity, but when modified to release granulocyte macrophage–colony-stimulating factor (GM-CSF), they were able to stimulate long-lasting anticancer immunological response [56]. Leveraging these insights, gene-transduced tumor cell vaccines (GVAX) are whole tumor cell-based vaccines collected from patients and cultured with granulocyte colony-stimulating factor (G-CSF) followed by transduction with adenoviral vector encoding GM-CSF [57]. The tumor cell product is then irradiated to arrest proliferation before autologous delivery to activate both adaptive and innate immune responses (Fig. 2).

In one study, five MDS patients received GVAX as five separate injections over three to four months as part of a phase I pilot study [58]. One patient showed hematological improvement (HI) and one patient attained transfusion independence [58]. A phase 1 clinical trial in MDS-RAEB and R/R AML displayed a good safety profile of this vaccine [59]; however, when compared with placebo in a phase 2 clinical trial, there was no survival benefit post-HSCT [57]. Interestingly, vaccines with higher GM-CSF secretion were associated with worse outcomes than vaccines with lower secretion of GM-CSF. suggesting that higher GM-CSF can, in some settings, paradoxically reduce effector immune responses.

One approach for increasing the efficacy of GM-CSF vaccines is through the use of adjuvants, such as lenalidomide. Lenalidomide is an approved treatment for MDS with 5q deletion and has been shown to enhance the immunological effect of vaccines in multiple myeloma [60]. A phase I study evaluated a bystander vaccine prepared by transfecting GM-CSF and CD40 Ligand into the K562 cell line and administered in combination with lenalidomide for MDS subtypes RAEB-1 (5–9% blasts in bone marrow) and RAEB-2 (10–19% blasts in bone marrow) [61]. This trial found that the vaccine was safe in intermediate and HR-MDS and CR was observed in 2/11 patients [61].

Overall, whole-cell vaccines have an advantage of targeting multiple genes and have a simpler manufacturing process. However, they could elicit off-target toxicity, and to date, response rates have been low.

## Dendritic cell vaccines

**Fig. 3:**
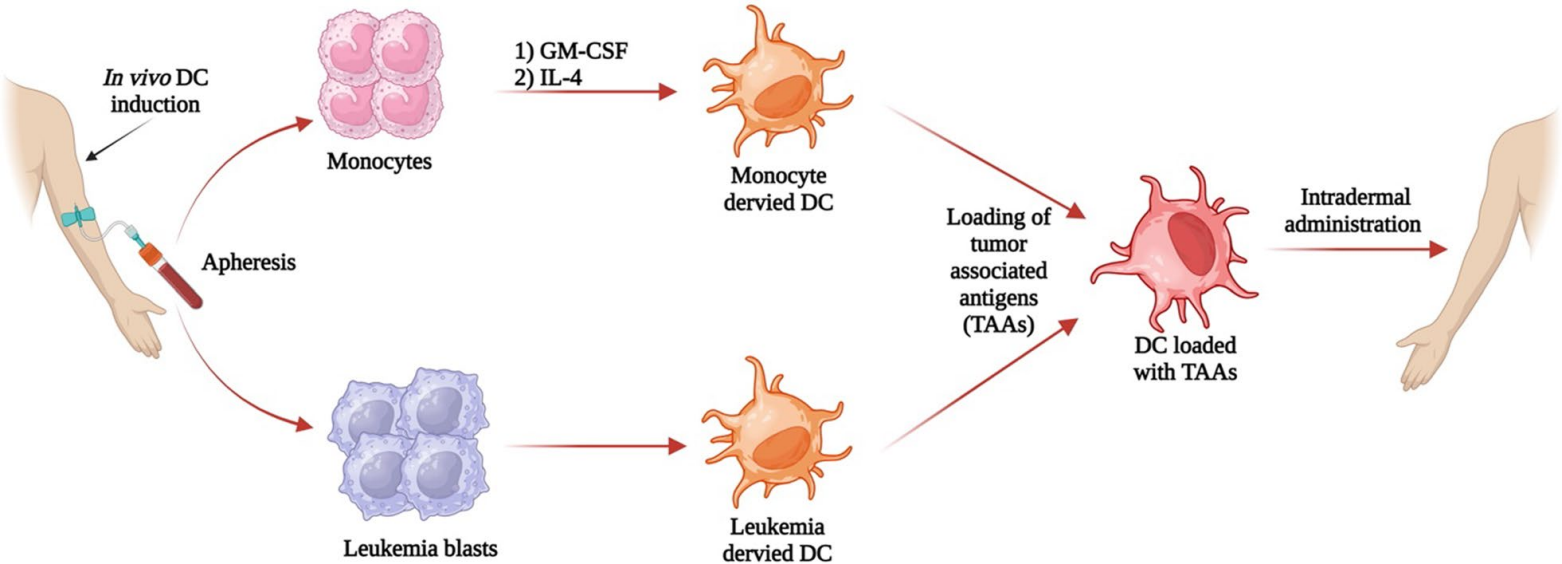
Schematic diagram of preparation and mechanism of action of dendritic cell vaccines

As pivotal immune players of the innate response, dendritic cells (DCs) are professional APCs that engage with MHC (I and II) molecules to trigger adaptive immunological responses and facilitate local inflammation. These immune cells can be produced ex vivo from allogeneic or autologous monocytes, CD34+ hematopoietic progenitor cells or leukemia-derived dendritic cells (DCleu) following leukapheresis [62]. Ambregner et al. proposed the use of immunomodulatory agents to initiate in vivo conversion of leukemic blasts to DCleu [1]. DCs can be loaded with peptides [63] apoptotic tumor bodies [64], viral vectors [65] or nucleic acids for expression of tumor targets on MHC-I/II before patient reinfusion [66]. Earlier in vitro studies showed that dendritic cells can present antigen and stimulate T-cell responses in MDS [67], validating their potential promise for hematological malignancies (Fig. 3).

A phase 1 trial for DCP-001, an allogeneic leukemia-derived DC vaccine, demonstrated increased duration of remission or smoldering disease in 7 out of 12 patients with intermediate- and high-risk AML/MDS who were not candidates for HSCT [68]. Responses were seen in patients who received vaccine immediately after achieving complete remission; this vaccine was produced by culturing myeloid leukemia cells in the presence of mitoxantrone to facilitate differentiation into DCleu and a phase 2 trial is currently active [69].

Although only a handful of dendritic cell vaccines studies have reached human testing in MDS, this strategy has considerable promise with more preclinical/clinical studies under investigation.

## Nanovaccines

Progress in nanomedicine has shed light on several promising applications of nanoparticles, including enhancing delivery and immunogenicity of cancer vaccines. A cell membrane-coated nanovaccine is a relatively new concept being evaluated in vivo studies. This consists of a nanoparticle core wrapped inside the antigen-rich cancer cell membrane. The strategy has been explored in breast cancer to potentiate the immunotherapeutic effect of immune checkpoint inhibitors and has demonstrated potent clinical response [70]. This is an especially feasible option for MDS since malignant blasts are readily obtainable from bone marrow aspirates as the source for antigen-loaded cancer cells. Johnson et al. successfully created AML cell membrane-coated nanoparticles (AMCNP) in which the NP core was bundled with CpG oligodeoxynucleotides to enhance efficacy of this multi-antigen and personalized vaccine [71]. This vaccine was able to elicit a significantly greater T-cell response than a control vaccine in mice models.

## Targeting the tumor microenvironment

The pathogenesis of MDS is largely unclear. Studies suggest that immune dysregulation and autoimmunity in early disease stages leads to bone marrow failure and proliferation of malignant myeloid precursors in later stages [5]′ [72]. This wide immune landscape and absence of distinct immune classification complicates immune targeting with available agents and development of vaccination strategies. For instance, immunosuppressive therapy (IST) exhibited promise in a phase 2 trial of MDS patients with hematological improvement in 33% patients, irrespective of IPSS categories [73]. However, a subsequent single-center retrospective study of IST found that, despite response rates similar to other standard therapies in low-risk disease patients, its effectiveness was somewhat limited in high-risk disease patients [74]. Therefore, it is important to prioritize the development and validation of immune classifications that can effectively guide the utilization of immune agents. While most IST studies have been focused on cytotoxic T-cells and natural killer cells historically, recent studies have elucidated the role of other immune players such as T-regulatory cells, MDSCs and dendritic cells (DCs).

MDSCs are immature myeloid cells that inhibit anti-tumor immunity by decreasing proliferation of cytotoxic T cells and promoting expansion of regulatory T cells in cancer patients. Kittang and colleagues identified a positive correlation between the number of MDSCs in peripheral blood with risk group in MDS [75]. They also found that MDSCs express CX3CR1 and CXCR4 which facilitate their migration to bone marrow [75]. As such, MDSC's significantly contribute to immune resistance mechanisms in MDS and may be a potential target to enhance efficacy of vaccination. Many targeted therapies designed to reduce their proliferation and mobilization to bone marrow have been proposed in clinical and preclinical studies in solid cancers, but this approach has not been explored in hematological malignancies [76]. A phase 1 MDS clinical trial evaluating SX-682, an oral agent with selective inhibition for chemokine receptors, C-X-C motif chemokine receptor 1 (CXCR1) and CX-C motif chemokine receptor 2 (CXCR2) is currently underway [77]. T regulatory cells are immune suppressor players of the immune system. Their role in MDS has been shown to evolve over the course of disease with decreased expression in early stages causing autoimmunity and increased expression in later stages causing tumor proliferation [78]. These recent findings could be utilized to target appropriate mechanisms in the pathogenesis of MDS and optimize the timing of immunotherapies. As the population in MDS is largely geriatric, vaccination in early stages can help in mitigating subsequent morbidity and mortality.

DCs are APCs that play a pivotal role in the immune system by capturing, processing and presenting antigens to T cells, thereby activating the adaptive immune response. However, in MDS, the function of DCs is impaired, leading to compromised immune surveillance and dysregulated immune responses [79]. Dendritic cell dysfunction in MDS involves abnormalities such as defective differentiation and maturation causing reduced numbers [80], impaired antigen processing leading to suboptimal activation of T cells [81], abnormal cytokine production and an immunosuppressive microenvironment due to increased levels of inhibitory factors and immune checkpoint molecules [5]. These factors hamper adequate immune response to vaccines; understanding the underlying mechanisms for dendritic dysfunction can help identify therapeutic targets (Table 1).

**Table 1:** Selected completed trials of MDS vaccines

Vaccine	Phase	N	NCT/ISRCTN	Adjuvants/additives	MDS Indication	Results across patient subsets
WT-1 peptide vaccine [28]	I	16	NCT00665002	montanide+ GM-CSF	HR-MDS	Well tolerated 1/2 patients (HR-MDS) with prolonged transfusion independence
WT-1 and PR-1 Peptide vaccine [24]	I	8	NCT00313638	montanide+ GM-CSF	MDS-RAEB; ineligible for HSCT	Well tolerated PR-1/WT-1-specific CD8+ T-cells
RHAMM Peptide vaccine [48]	I/II	10	ISRCTN32763606	Incomplete Freud adjuvant+ GM-CSF	MDS < 20% BM blasts (RA, RAEB-1, RAEB-2), MM	RHAMM-specific T-cell responses 1 MDS patient with blast reduction and transfusion-independent
DEC-205/NY-ESO-1 fusion Peptide vaccine [42, 118]	I	9	NCT01834248 NCT03358719	Decitabine Addition of nivolumab	Intermediate and HR-MDS	NY-ESO-1-specific T cell responses No results posted
NY-ESO-1, MAGE-A3, PRAME, WT-1 Peptide vaccine [46]	I	5	NCT02750995	Azacitidine	HR-MDS	All patients progressed to AML; mean time to progression of 4.9 months from inclusion in the study
Whole-cell endogenous tumor antigen dendritic cell vaccine [68]	I	12	NCT01373515	DCP-0001 vaccine	HR-MDS	7/12 patients with positive response (median OS 1090 days); 5/12 with progressive disease (Median OS 144 days)
TAA whole-cell vaccine [61]	I	11	NCT00840931	K562-GM-CSF-CD40L vaccine+ lenalidomide+ GM-CSF	Intermediate and HR-MDS (failed HMA treatment)	Well tolerated CR in 2/11, marrow CR in 1/11, PR in 1/11
TAA whole-cell vaccine [57]	II	15	NCT01773395	GVAX vaccine	MDS-RAEB	No significant difference in 18-month PFS, OS and relapse incidence between GVAX vs placebo
PR-1 Peptide vaccine [36]	I/II	62 (11 MDS)	NCT00004918	Montanide+ GM-CSF	MDS-RAEB	Clinical response (CR, PR and hematological improvement) in 24% patients

While other potential targets [82], such as cytotoxic T-lymphocyte antigen-4 (CTLA-4) and programmed death receptor-1 (PD-1), have demonstrated strong anti-tumor effects in solid tumors, their therapeutic potential in myeloid malignancies has been limited, perhaps due to lower microsatellite instability in MDS [83]. Evidence shows that cancer types with higher mutational burdens respond better to T cell-based therapies and checkpoint inhibition [84]. In MDS, mutational burden correlates with disease severity, but it is considerably lower than in most other cancers [4, 85]. In contrast to bladder cancer and melanoma, where T cell reactivity was detected in 31 out of 42 individuals, a study of MDS patients found T cell responses in only two out of 13 subjects [86]. This suggests that generating an immunogenic neoepitope is rare for MDS patients and might necessitate new approaches for effective neoantigen-mediated tumor recognition. While specific T cells are scarce in MDS, combining checkpoint inhibitors with epigenetic modulating agents such as DNA methyltransferase inhibitors, they may enhance T cell reactivity against upregulated antigens, like cancer–testis antigens [87].

CD47, a macrophage checkpoint, has been identified as highly expressed on myeloid leukemia stem cells and acts as a ligand for signal regulatory protein alpha (SIRPα) found on macrophages [88]. Upon activation, this interaction inhibits phagocytosis. Promising results have been observed with magrolimab, an anti-CD-47 antibody, in combination with AZA for AML and HR-MDS treatment [3]. Additionally, evorpacept, an engineered fusion protein with a high affinity for blocking CD47, is currently undergoing phase 2 trials for HR-MDS [89].

Another potential target for T-cell checkpoint therapy is T-cell immunoglobulin domain and mucin domain-3 (TIM-3), which has been identified for its selective expression on leukemia stem cells and blasts, promoting self-renewal [90]. TIM-3 expression in MDS increases during disease progression and AML transformation [90]. Sabatolimab, an anti-TIM-3 antibody, has shown long-lasting responses when combined with HMAs in newly diagnosed AML and HR-MDS patients [91]. There is a potential for combining these immunotherapeutic agents with vaccines to enhance their efficacy (Table 2).

**Table 2:** MDS vaccines in ongoing trials

Vaccine	Payload	Phase	NCT	MDS Indication	Product(s)
Dendritic cell vaccine [119]	WT-1	I/II	NCT03083054	HR-MDS	WT-1 mRNA electroporated in autologous DCs
Peptide vaccine [120]	IL-3	I	NCT03113643	HR-MDS	Diphtheria toxin linked with IL-3+ AZA or AZA/venetoclax
Dendritic cell vaccine [121]	Whole cell	I	NCT04999943	Elderly (> 60 years) MDS	DC vaccine+ HMA

In addition to the immune system regulators mentioned above, several surface molecular targets are currently being investigated which may have potential role in vaccine strategies for MDS [92]. CD123, which forms the alpha-chain of IL-3 receptor expressed on cell membrane of myeloid progenitor cells, is one such target [93]. In a Phase 1B study of APVO436, a humanized bispecific antibody targeting both CD123 on leukemia blasts and CD3 on T cells to trigger T cell cytotoxicity against leukemia cells, marrow CRs were observed in three out of six assessable patients with high-risk MDS [94]. CD33 is another potential target, which is expressed on the surface of leukemia blast cells and MDSCs [92]. Gemtuzumab ozogamycin (GO) is a humanized monoclonal antibody targeting CD33 approved for the treatment of CD33+ AML patients [95]. Multiple clinical trials are examining the potential of GO in combination treatments for MDS. A phase II study, which investigated the combination of decitabine and GO, did not reveal a survival benefit in high-risk MDS patients [96]. However, an ongoing study investigating the combination of GO with CPX-351 has shown a clinical response in one out of two high-risk MDS patients [97].GTB-3550 Trike, a Tri-Specific Killer Engager targeting CD16/IL-15/CD33 is currently undergoing investigation in a phase 1 trial [98]. TLR2, a surface molecule belonging to Toll-like receptor family, is shown to be overexpressed in CD34+ cells in the bone marrow, leading to impaired innate immune function due to the dysregulation of the IL-8 pathway [99]. Tomaralimab, a monoclonal antibody against TLR2, has shown promising results in phase I/II clinical trial involving patients with low- and intermediate-1-risk MDS [100]. While preclinical studies have identified CD99 and IL1RAP (IL-1 receptor accessory protein) as potential therapeutic targets in high-risk MDS, clinical investigation is currently lacking [101, 102].

## Advancements in cancer vaccine platforms: an evolutionary perspective

The major cancer vaccine platforms include peptide-based, cell-based and nucleic acid-based (Table 3). Insights gained from numerous preclinical and clinical studies has led to development of several innovative vaccine development strategies. Table 4 provides an overview of key technologies in the field of cancer vaccine development. The selection of appropriate antigens and the optimization of delivery systems play pivotal roles in ensuring comprehensive CTLs and T helper cell responses [103]. Advances in genome sequencing have led to the identification of neoantigens, eliminating self-tolerance immune mechanisms associated with tumor-associated antigens [104, 105]. Neoantigens represent newly formed antigens that arise as a consequence of tumor-specific alterations [106]. In the context of peptide vaccines, it has been observed that short peptides lack the ability to generate robust and enduring immunogenic responses due to suboptimal antigen presentation, insufficient activation of helper T cells and a short half-life [107]. Conversely, vaccines developed from whole proteins have struggled to provide clinical benefits due to poor processing and presentation by APCs. This understanding has led to the development of long synthetic peptides, exhibiting improved immunogenic responses through APC-mediated degradation by endosomal pathway and optimal activation of T helper cells [108]. Another strategy involves combining these peptides with carrier proteins, such as heat shock proteins (HSP), to enhance antigen presentation and CD8+ T cell responses, although this approach has not been explored in MDS and demonstrated little success in other cancers [109]. Adjuvants, including nanoparticles, cytokines and Toll-like receptor (TLR) ligands [110], are commonly incorporated into peptide vaccines to enhance their efficacy [107]. Nanoparticles, in particular, serve as effective delivery systems, preventing protein degradation [111]. Notably, the TLR3 agonist polyinosinic–polycytidylic acid (poly I: C) induces a robust Th1 response, enhancing vaccine efficacy [110]. Cytokines such as IL-2, GM-CSF and interferon (IFN) are frequently tested adjuvants with promising results.

**Table 3:** Overview of cancer vaccine platforms

Vaccine type	Merits	Drawbacks	Tested MDS vaccines
Peptide-based vaccine	Low toxicity Easy production and low cost	Low immunogenicity Short peptides are HLA-restricted	WT4869 DSP-7888 PR-1 vaccine NY-ESO-1 vaccine
Cell-based vaccine: 1. Whole-cell vaccine 2. Dendritic cell vaccine	Broader target population DCs are potent APCs	Nonspecific targets Potential release of immunosuppressive factors Cumbersome manufacturing process and high cost Immature DCs can induce tolerance	GVAX DCP-001
Nucleic acid-based vaccine (DNA and mRNA)	Easy production Encode multiple antigens No HLA restriction	Require delivery systems Difficult handling and storage	Not tested

**Table 4:** Major cancer vaccine technologies

Cancer vaccine technology	Description	Manufacturing steps	Key quality control checks
Peptide-based vaccines	Utilize specific peptides derived from tumor-associated antigens to stimulate an immune response	Antigen selection and characterization Peptide design Peptide synthesis and purification Formulation with adjuvants Sterilization	Peptide identification and purity testing Potency Stability Interaction with adjuvant
Neoantigen-based vaccines	Target neoantigens unique to an individual’s tumor cells to activate personalized and targeted immune response	Tumor sample collection Genomic and proteomic analysis to identify neoantigens Production of neoantigen peptides or neoantigen encoding genetic material Formulation with adjuvants or carrier proteins	Identity and purity testing Potency Sterility Stability
Dendritic cell vaccines	Utilize DCs as professional antigen-presenting cells to activate immune system	Patient sample collection and isolation of DCs Dendritic cell culture and maturation Loading dendritic cells with tumor antigens Formulation with adjuvants Sterilization	Assessment of dendritic cell viability and expression of maturation markers Potency Stability
Whole-tumor-cell Vaccines	Utilize whole tumor cells to stimulate a broad immune response	Patient sample collection and isolation of tumor cells Inactivation and modification of tumor cells Formulation with adjuvant Sterilization	Identity and purity testing Sterility Potency Stability
mRNA Vaccines	Introduce tumor antigen encoding mRNA o activate immune response against tumor antigens	Antigen selection and design of mRNA sequence Synthesis of mRNA using in vitro transcription Purification of mRNA 3’End Capping and Polyadenylation Formulation with lipid nanoparticles or protein-based carriers	Identity and purity testing of mRNA sequence Sterility Potency Stability
DNA Vaccines	Introduce tumor antigen encoding DNA to activate the immune response against tumor antigens	Selection of TAA Cloning the genes encoding TAA into a plasmid vector Plasmid amplification and purification Formulation with delivery vectors Sterilization	Identity, purity and quantification testing of purified plasmid DNA Sterility
Viral vector-based vaccines	Utilize viral vector to deliver genetic material encoding tumor antigens	Selection of viral vector Synthesis of recombinant viral vector Propagation in host cell followed by harvesting and purification Formulation with adjuvant Sterilization	Identity and purity testing Sterility Potency Quantitative analysis of viral vector Host cell DNA residual testing Stability
Virus like particle (VLP)-based vaccine	Use structural mimicry of viruses to generate robust immune response	Propagation of genes encoding tumor-associated antigen in host cells Identification of suitable expression system for production of VLP Fusion or insertion of TAA genes in expression system and production of TAA modifies VLPs Harvesting and purification Formulation with stabilizers and adjuvants Sterilization	Identity, purity and quantification of TAA-modified VLPs Sterility Stability

In the realm of cell-based vaccines, various advancements have contributed to enhancing their efficacy. Whole-cell vaccines, employing a straightforward approach to target a broad array of CTL and T helper cell epitopes, have demonstrated low immunogenicity thus far. The addition of immune stimulants like GM-CSF and IL-2 can enhance immune responses [56, 112]. Adenoviral integration has proved to be an effective method for stimulating endogenous GM-CSF production in the GVAX vaccine [57]. Conventionally, DC vaccines are derived from monocytes and undergo extensive ex vivo culturing, potentially diminishing the immunogenic potential of the vaccine. A newer approach involves extracting patient-derived circulating DCs, showing improved clinical results [113]. The adoption of antibody-coated magnetic beads has allowed faster and more efficient native DC isolation [114].

Overcoming challenges in nucleic acid-based vaccine design involves addressing constrained uptake by APCs due to degradation and low transfection efficiency. Novel methods such as electroporation, nanoparticles [115], gene guns, liposomal delivery systems and microneedle arrays have been successful in overcoming these limitations [116]. The gene gun method involves loading DNA/RNA onto nanoparticles coated with heavy metals, facilitating their entry into APCs [115]. A phase 2 study investigating mRNA-electroporated DC vaccine in AML patients showed a 43% clinical response rate and improved OS in responders [117].

## Conclusion and areas of future research

Collectively, therapeutic vaccines for MDS have shown acceptable safety profiles, but robust clinical responses have not yet been consistently observed despite induction of robust immunological responses. A significant cytotoxic immune response capable of eliminating stem cells is likely required for a vaccine to be effective [8]. Robust immunological responses are dependent on a well-functioning innate immunity and functional APCs may be inadequate in MDS patients where DCs may be inherently dysfunctional with lower precursor frequencies. This is a significant obstacle that must be overcome.

Combining vaccines with immunotherapeutic agents have the potential to create a synergistic effect, leading to improved outcomes for patients. By blocking certain inhibitory pathways or enhancing immune cell activity, these agents can bolster the immune responses induced by vaccines. Combinations of vaccines with other immunotherapies such as chimeric antigen receptor T cell therapy (CAR-T), bispecific antibodies and immune checkpoint inhibitors are increasingly being considered for other hematological malignancies; however, the efficacy of these approaches has not yet been demonstrated in AML/MDS. Since agents targeting MDSCs, CD47 or TIM-3 may synergize with vaccine immunotherapy, larger comparative studies are needed to ascertain benefit [8].

The advanced stages of MDS are likely to be less ideal for inducing an immune response. More ideal settings might include lower tumor burden, either early in the course of disease or for advanced disease, following cytoreduction when the disease is in remission or after allogeneic hematopoietic stem cell transplantation (HSCT). Caution should be ensured in the setting of post-HSCT as vaccination may alter immunity in such a way as to increase the risk for graft versus host disease (GVHD). Vaccines developed from HSCT donor-derived DCs are promising strategies currently being evaluated in trials [14]. Equally attractive might be the use of vaccines in early MDS to prevent progression. Continued progress in transgenic mouse models to simulate the unique biological and molecular features may aid in validating promising agents and optimizing the timing for vaccination strategies in MDS [15]. Larger confirmatory clinical trials, ideally with a comparison arm, are required to confirm clinical efficacy. Early insights from vaccine studies with safety and activity across MDS cohorts promises new immunotherapeutic advances for patients battling this difficult disease.

## Abbreviations

AML: Acute myeloid leukemia
APC: Antigen-presenting cells
AZA: Azacitidine
CAR-T: Chimeric antigen receptor T cell therapy
CLL: Chronic lymphocytic leukemia
CML: Chronic myeloid leukemia
CMML: Chronic myelomonocytic leukemia
CR: Complete remission
DC: Dendritic cell
DCleu: Leukemia-derived dendritic cell
GVAX: Gene-transduced tumor cell vaccine
GVHD: Graft versus host disease
GM-CSF: Granulocyte macrophage–colony-stimulating factor
HI: Hematological improvement
HSCT: Hematopoietic stem cell transplantation
HR-MDS: High-risk myelodysplastic neoplasms
HLA: Human leukocyte antigen
HMA: Hypomethylating agents
IPSS: International Prognostic Scoring System
LAA: Leukemia-associated antigens
LR-MDS: Low-risk myelodysplastic neoplasms
MAGE-A3: Melanoma antigen family A
MM: Multiple myeloma
MDSCs: Myeloid-derived suppressor cells
MDS: Myelodysplastic neoplasms
MHC: Major histocompatibility complex
OS: Overall survival
PRAME: Preferentially expressed antigen of melanoma
PD-1: Programmed cell death protein-1
PFA: Progression-free survival
PR-1: Proteinase-3+ neutrophil elastase
PR: Partial remission
RHAMM: Receptor for hyaluronic acid-mediated motility
RA: Refractory anemia
RAEB: Refractory anemia with excess blasts
R/R AML: Relapsed or refractory acute myeloid leukemia
TIM-1: T-cell immunoglobulin and mucin-1
TAA: Tumor-associated antigens
WT-1: Wilms tumor 1
